# Cardioprotection by Cocoa Polyphenols and *ω*-3 Fatty Acids: A Disease-Prevention Perspective on Aging-Associated Cardiovascular Risk

**DOI:** 10.1089/jmf.2018.0002

**Published:** 2018-10-16

**Authors:** Sergio Davinelli, Graziamaria Corbi, Stefano Righetti, Barry Sears, Hector Hugo Olarte, Davide Grassi, Giovanni Scapagnini

**Affiliations:** ^1^ Department of Medicine and Health Sciences “ V. Tiberio”, University of Molise, Campobasso, Italy.; ^2^ Department of Cardiology, San Gerardo Hospital, Monza, Italy.; ^3^ Inflammation Research Foundation, Peabody, Massachusetts, USA.; ^4^ CasaLuker S.A., Bogotà D.C., Bogotà, Colombia.; ^5^ Department of Life, Health and Environmental Sciences, University of L'Aquila, L'Aquila, Italy.

**Keywords:** *cardiovascular disease*, *functional food*, *inflammation*, *nutrition*, *oxidative stress*, *prevention*, *supplementation*

## Abstract

Cardiovascular disease (CVD) remains the leading cause of death today. Many of the biochemical alterations associated with the pathophysiology of CVD can be modified by adequate intakes of bioactive nutrients through a correct diet or supplementation. Recently, there has been growing public and clinical interest in cocoa polyphenols (CPs) and omega-3 (*ω*-3) fatty acids. A plethora of nutritional intervention trials and experimental studies demonstrates that consumption of these bioactive food compounds is beneficial to promote cardiovascular health. The purpose of this review is to summarize the major cardioprotective effects of CPs and *ω*-3 fatty acids, providing a scientific rationale for incorporating the combination of these molecules as a nutritional intervention in the prevention of CVD. Although several studies have shown the individual cardioprotective nature of these compounds, a combination treatment with CPs and *ω*-3 fatty acids may be a promising approach to enhance the preventive value of these molecules and reduce cardiovascular risk factors associated with aging. Therefore, this article also reviews some of the key studies on the interaction between CPs and the metabolism of *ω*-3 fatty acids.

## Introduction

Cardiovascular disease (CVD) is the leading cause of death today. Across the world, CVD results in 1 in 3 deaths per year and more than half of the deaths related to CVD occurs in individuals aged 65–74 years.^1^ The economic load amounts to almost €170 billion annually in Europe and over $190 billion per year in the United States.^2,3^ Cardiovascular aging includes atherosclerosis, coronary artery disease, hypertension, heart failure, and atrial fibrillation. Aged hearts are characterized by reduced contractility, impaired diastolic function, and atrium dilatation. Morphological changes that result in cardiac aging include calcification and cholesterol-rich plaque formation, in addition to defective relaxation and endothelial dysfunction. These alterations of the heart associated with aging occur in both the vasculature and the myocardium.^4,5^ Moreover, the key molecular and cellular mechanisms underlying cardiovascular aging involve oxidative stress, mitochondrial dysfunction, age-related low-grade inflammation, increased apoptosis, cellular senescence, reduced bioavailability of nitric oxide (NO), impaired bioenergetic efficiency, age-related decline of autophagy, and activation of the renin–angiotensin–aldosterone system.^6,7^ The major risk factor for CVD is advancing age, however, healthy lifestyle and diet have the potential to reduce CVD risk. Research on dietary interventions typically focuses on reducing one or more traditional CVD risk factors such as systolic blood pressure (BP) and low-density lipoprotein (LDL) cholesterol. Moreover, several studies showed how identification of the active constituents of specific dietary patterns is crucial in the formulation of appropriate dietary guidelines.^8,9^

## Health Benefits of Cocoa and Omega-3 Fatty Acids

**FIG. 1.**
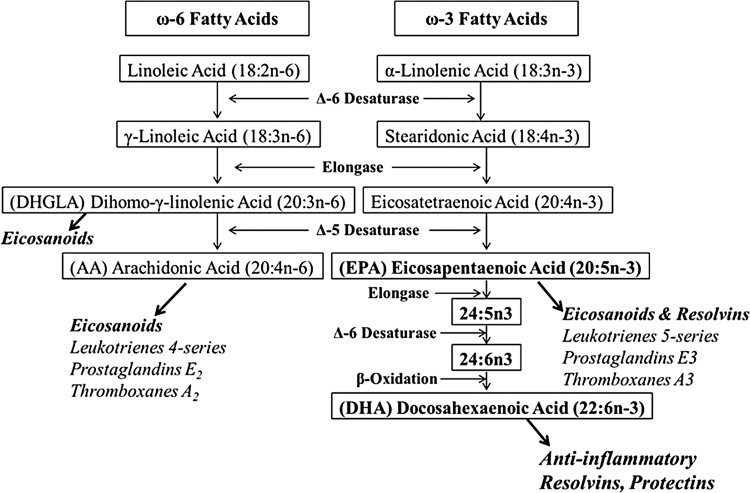
PUFA and eicosanoid biosynthesis. ALA (18:3n-3) and LA (18:2n-6) are PUFAs obtained from the diet. Mammals convert LA and ALA to LC fatty acids using a series of desaturation and elongation reactions. Relevant intermediates to synthesize EPA (20:5n-3), DHA (22:6n-3), and AA (20:4n-6) include stearidonic acid, eicosatetraenoic acid, *γ*-linolenic acid, and DGLA (20:3n-6). AA and EPA are substrates for the synthesis of eicosanoid products such as PG and LT. EPA and DHA are also metabolized to resolvins and protectins, which are very potent anti-inflammatory mediators. The products of *ω*-6 fatty acids tend to promote inflammation, while the products of LC *ω*-3 fatty acids have anti-inflammatory properties. The LC *ω*-3 fatty acids may reduce the risk of CVD by disrupting the biosynthesis of AA-derived inflammatory eicosanoids. AA, arachidonic acid; ALA, *α*-linolenic acid; CVD, cardiovascular disease; DGLA, dihomo-*γ*-linolenic acid; DHA, docosahexaenoic acid; EPA, eicosapentaenoic acid; LA, linoleic acid; LC, long chain; LT, leukotrienes; PUFAs, polyunsaturated fatty acids; *ω*-3, omega-3.

Many of the biochemical alterations associated with the pathophysiology of CVD can be modified by adequate intakes of bioactive nutrients through a correct diet or supplementation.^10–12^ In this context, functional foods and dietary supplements may exert a positive effect on the prevention of CVD. There is a great number of bioactive compounds with a wide spectrum of biological properties affecting heart and cardiovascular system. Over the past 10 years, researchers have become increasingly interested in cocoa polyphenols (CPs) due to their potent antiradical properties. Recent nutritional intervention trials and molecular studies demonstrate that consumption of cocoa, particularly rich in flavanols, is beneficial to promote cardiovascular health.^13^ For example, flavanol-rich cocoa decreases the plasma level of F_2_-isoprostanes, markers of lipid peroxidation, and therefore, increases total antioxidant activity.^14^ Among the wide variety of functional foods, there is growing public and clinical interest in marine fish oil and its high content of long-chain (LC) omega-3 (*ω*-3) fatty acids. Several epidemiologic studies suggested a reduced risk of CVD associated with fish oil consumption. Dietary LC *ω*-3 polyunsaturated fatty acids (PUFAs) have strong anti-inflammatory properties and their beneficial effects have been reported in pathologies and conditions associated with inflammation, including CVD. The LC *ω*-3 fatty acids showed improvement in systolic and diastolic BP and reduced inflammation within the artery wall. These compounds can also decrease the incidence of heart attack and/or stroke.^15^ The molecular basis of *ω*-3 fatty acid action was not established until recently. The *ω*-3 PUFAs compete with the storage of arachidonic acid (AA), replacing it and blocking the production of proinflammatory eicosanoids. Indeed, the family of eicosanoids is synthesized from AA by the initial activities of either cyclooxygenases (isoforms COX1 or COX2) or lipoxygenases (LOX) and downstream enzymatic reactions. There are several main classes of eicosanoids: prostaglandins (PG), thromboxanes (Tx), leukotrienes (LT), and lipoxins (Lx). The *ω*-3 PUFAs displace AA in membrane phospholipids, reducing the production of AA-derived eicosanoids, while increasing those (resolvins and protectins) generated from *ω*-3 PUFAs (Fig. 1). This altered eicosanoid profile might influence inflammation, thrombosis, and vascular function.^16^ In this article, we highlight that a feasible combination of CPs and LC *ω*-3 fatty acids may positively influence detrimental processes underlying cardiovascular aging. To the best of our knowledge, a review focusing on these compounds and the possible synergistic effects of CPs and LC *ω*-3 supplementation on cardiovascular aging has not been performed. Therefore, the purpose of this review is to summarize the major cardioprotective properties of CPs and LC *ω*-3 fatty acids to highlight how their combined use may be a promising preventive approach in CVD. The benefits of a combination treatment with these two dietary compounds will be also discussed.

## Search Strategy

The following question guided this review: “Is the combination of CPs and LC *ω*-3 fatty acids more effective in affecting cardiovascular system than CPs or LC *ω*-3 fatty acids alone?” The literature search was carried out referring to PubMed, using predetermined keywords and a combination of Mesh terms. The search terms, combined with Boolean operator AND/OR, related to the aspects considered in this review were: (1) CVD: coronary artery, atherosclerosis, ischemic heart, ischemic, ischemia, cerebral stroke, cerebrovascular, CVD, cardioprotection, cardioprotective, cardiovascular prevention, cardiovascular aging; (2) Food/diet: cocoa, chocolate, dark chocolate, polyphenols, cocoa flavanols, cocoa flavonoids, cocoa catechins, polyunsaturated fatty acids, PUFA, PUFA metabolism, LC PUFA, *ω*-3 PUFA, n-3 PUFA; *ω*-6 PUFA, n-6 PUFA, *ω*-6/*ω*-3 ratio, n-6/n-3 ratio. We then focused our attention on studies in English only, full length, performed on humans, *in vitro* or animal studies.

## Cardioprotective Actions of Cps

Cocoa is recognized as a rich source of polyphenols. Total polyphenol content of the cocoa beans is about 6–8% by dry weight and black chocolate is considered one of the major source of antioxidants.^17,18^ The most predominant phenolic phytochemicals in cocoa are flavonoids, which include mainly flavonols, flavanols, flavanones, isoflavones, and nonflavonoids.^18,19^ Catechins, anthocyanidins/anthocyanins, flavonol glycosides, and procynanidins are the major polyphenolic compounds in cocoa.^20^ Table 1 provides a list of the main polyphenols found in cocoa. The molecular mechanisms of cocoa phytochemicals associated with their potential beneficial effects on human health have been reviewed extensively elsewhere.^21^ The studies on the cardiovascular effects of cocoa highlight that CPs mediate an increase of the bioavailability of NO in the endothelium and reverse endothelial dysfunction in CVD. NO from endothelium prevents leukocyte adhesion and migration, smooth muscle cell proliferation, platelet adhesion, and aggregation. CPs reduce vascular arginase activity in endothelial cells, which increases the levels of L-arginine used to synthesize NO by endothelial NO synthase (eNOS). Accordingly, increased NO bioavailability induced by CPs promotes a relaxation of vascular smooth muscle cells, leading to vasodilation.^22^ Besides their direct effects on eNOS activity, cocoa flavanols and procyanidins exert strong antioxidant effects. In general, bioactive compounds with antioxidant properties have been studied extensively to assess the effects on disease states such as CVD. For example, oxidation of LDL plays a crucial role in the formation of atherogenic plaques and numerous antioxidants prevent LDL oxidation and delay atherogenesis in animal models. However, a comprehensive Cochrane review of the impact of antioxidant supplements (administered singly or in various combinations) on healthy individuals (26 trials), or in people with one or more of a range of diseases (52 trials), including CVD (10 trials), found no overall reduction in all-cause mortality, irrespective of the combination of antioxidants used.^23^ Nevertheless, several studies have been performed to determine the role of CPs in lipid oxidation. Protection of LDL particles against oxidation by cocoa flavanols is a crucial mechanism accounting for the cocoa antioxidant property and to reduce atherosclerotic progression.^24,25^ Of note, cocoa flavonoids interact with myeloperoxidase, an enzyme involved in peroxidation of LDL. The myeloperoxidase-mediated peroxidation of LDL was blocked by CPs.^26^ Other investigators observed that CPs have immunoregulatory effects and reduce the production of reactive oxygen species in activated leukocytes.^27^ Moreover, data from numerous studies suggest that cocoa flavanols can modify the inflammatory process and thus reduce cardiovascular risk.^28^ Long-term exposure to proinflammatory cytokines such as tumor necrosis factor alpha (TNF-*α*) or interleukin 6 (IL-6) is detrimental to the myocardium and associated with chronic heart failure.^29,30^ Nuclear factor-kappa *β* (NF-*κβ*) is the major inducible transcription factors controlling the inflammatory response and the transcription of several proinflammatory cytokines. The modulation of the NF-*κβ* signaling is important in the reduction of the inflammatory response and may represent a common pathway to all cocoa anti-inflammatory effects. Experimental evidence has demonstrated that cocoa epicatechin, catechin, and flavanols reduce NF-*κβ* activation, thereby reducing production of proinflammatory cytokines and oxidative burst.^31,32^

**Table 1.** Major Cocoa Polyphenols

*Class*	*Compounds*
Flavanols	(−)-Epicatechin
	(+)-Catechin
	(−)-Epicetechin-3-*O*-gallate
	(−)-Epigallocatechin
	Procyanidin B1 (epicatechin-(4*β*(8)-catechin)
	Procyanidin B2 (epicatechin-(4*β*(8)-epicatechin)
	Procyanidin B2-*O*-gallate (epicatechin-3-*O*-gallate-(4*β*(8) epicatechin)
	Procyanidin B2-3,3-di-*O*-gallate (epicatechin-3-*O*-gallate-(4*β*(8)-epicatechin-3-*O*-gallate)
	Procyanidin B3 (catechin-(4*β*(8)-catechin)
	Procyanidin B4 (catechin-(4 *β*(8)-epicatechin)
	Procyanidin B4-3-*O*-gallate (catechin-(4*β*(8)-epicatechin-3-*O*-gallate)
	Procyanidin C1 (epicatechin-(4*β*(8)-epicatechin-(4b(8)-epicatechin)
Flavonols	Quercetin
	Quercitin-3-*O*-arbinoside
	Quercitin-3-*O*-galactoside
	Isoquercitin
Anthocyanins	3-alpha-L-Arabinosidyl cyanidins
	3-beta-D-Galactosidyl cyanidins
Flavones	Luteolin
	Luteolin-7-*O*-hyperoside
	Iso-orientin
	Vitexin
Flavanones	Naringenin
	Naringenin-7-*O*-glucoside
Phenolic acids	Chlorogenic acid
	Vanillic acid
	Coumaric acid
	Phloretic acid
	Caffeic acid
	Ferulic acid
	Phenylacetic acid
	Syringic acid

## Cardiovascular Benefits of Cocoa From Human Studies

Epidemiological studies demonstrated that a diet rich in CPs reduces cardiovascular events in the general population. The Kuna Indians, a population living on the island off the Panama coast, have a diet characterized by high intake of cocoa and a lower BP compared to other Pan-American populations. The Kuna Indians have low incident of heart attack and stroke but after migration to an urban city they change their diet and this cardioprotection is lost.^33^ The Iowa Womens Health Study reported an inverse relationship between chocolate intake and mortality from coronary heart disease in postmenopausal women.^34^ The European Prospective Investigation into Cancer and Nutrition observed a lower rate of myocardial infarction and stroke, after 8 year of follow-up, in subjects who consumed 7.5 g/day of chocolate.^35^ Also the Stockholm Heart Epidemiology Program reported a lower cardiac mortality associated with higher chocolate intake in nondiabetic patients with previous myocardial infarction.^36^ Furthermore, several clinical trials have investigated the effects by which CPs potentially reduce CVD risk, improving lipid peroxidation, BP, inflammation, lipid metabolism, and glucose metabolism.^37–40^ Studies on the effects of CPs have shown that consumption of cocoa decreases LDL oxidation and lipid peroxidation biomarkers such as F_2_-isoprostane, among healthy subjects.^14,41,42^ Although the effect of cocoa products on lipid changes is equivocal, Grassi *et al.* reported that consumption of dark chocolate reduces serum LDL cholesterol concentrations in hypertensive subjects.^43^ In response to CPs consumption, it has also been shown an increase in plasma high-density lipoprotein (HDL) cholesterol and a decrease in plasma triglyceride.^41,44,45^ Experimental data from rats showed a specific hypocholesterolemic effect of catechins.^46^ In addition, various studies have confirmed the role of flavonoids as antioxidants in biological systems. Flavonoids in chocolate have been shown to exert potent antioxidant effects *in vitro* assays under artificial oxidative stress and increase in antioxidant capacity as part of various chocolate feeding trials.^47^ Additionally, because lipid soluble flavonoids may intercalate into the membranes of lipoprotein particles, flavonoids may reduce lipid peroxidation of biological membranes.^46,47^ Nevertheless, a meta-analysis including 102 trials on HDL outcomes, and 92 on LDL outcomes, reported that chocolate, cocoa, and flavonoids consumption had no effect on LDL concentrations.^37^ Therefore, even though studies in both rats and humans reported that cocoa/chocolate consumption and intake of polyphenols suppressed serum LDL cholesterol concentrations and susceptibility of LDL to oxidation and increased HDL cholesterol levels, most of the studies showed a neutral effect on lipid profile. Cocoa and chocolate contain a high level of fats. Indeed, cocoa butter derived from cocoa plants and predominantly found in dark chocolate contains an average of 33% of oleic acid (cis-18:1 monounsaturated), 25% of palmitic acid (16:0 saturated), and 33% of stearic acid (18:0 saturated).^48^ In particular, several studies have suggested that stearic acid may be noncholesterolemic.^48,49^ A meta-analysis of 60 controlled feeding trials concludes that stearic acid neither lowers HDL, nor increases LDL or total cholesterol.^50^ This study also suggests that per 1% energy isocaloric replacement of stearic acid for carbohydrates, stearic acid ingestion significantly lower serum triglycerides by 17.0 nmol/L. A more recent meta-analysis, aiming to evaluate the cardiometabolic effects of cocoa flavonoids (cocoa flavanols ranged from 166 to 2110 mg/d, and intervention duration ranged from 2 to 52 weeks), showed that cocoa flavanol intake significantly improved insulin sensitivity and lipid profile.^51^ Furthermore, results from intervention studies reported that the intake of dark chocolate was associated with decreased systolic BP in healthy subjects, and this effect has also been observed in hypertensive subjects and glucose intolerant hypertensive patients.^52,53^ Recently, by applying the Framingham Risk Score, the Flaviola Health Study reported that cocoa flavanols improve endothelial function. In healthy individuals, regular cocoa flavanols intake improved cardiovascular biomarkers, demonstrating that dietary flavanols have the potential to maintain cardiovascular health even in low-risk subjects.^54^ Cocoa flavanols have also a strong anti-inflammatory properties *in vitro* but recent human studies reported a modest support to the anti-inflammatory effect of cocoa.^55^ However, even though little clinical evidence exists that consumption of CPs may reduce inflammation, a randomized clinical trial with proanthocyanidine-rich cocoa powder demonstrated a reduction in plasma markers of inflammation in adults with high CVD risk.^56^ Some studies suggest that cocoa flavonoids may inhibit platelet aggregation by downregulating cellular eicosanoid synthesis.^57–59^ Eicosanoids are lipidic products involved in the regulation of the vascular tone and recruitment of immune cells into the vascular wall. In particular, eicosanoids such as PGE2, TxA2, and LT4 are derivatives of the AA and mediators involved in the inflammatory response. Despite the interest and the crucial role that CPs may exert as inhibitors of platelet aggregation, there is no a comprehensive study investigating the relationship between CPs, inhibition of platelet aggregation, and downregulation of eicosanoid synthesis. Therefore, to clarify the antiplatelet action of CPs more studies are needed.

## Consumption of LC *ω*-3 Fatty Acids and Cardiovascular Prevention

The growing interest LC *ω*-3 (or n-3) fatty acids was triggered by epidemiologic studies conducted with Greenland Eskimos.^60^ LC PUFAs of the *ω*-3 series are essential dietary compounds because mammals cannot synthesize them. Of note, within the family of PUFAs there is also a different group belonging to the *ω*-6 series. LC PUFAs can only be synthesized by mammals from the C18 precursors of each PUFA family, namely LC *ω*-6 fatty acids from linoleic acid (LA, C18:2n-6) and LC *ω*-3 fatty acids from *α*-linolenic acid (ALA, C18:3n-3). The LC *ω*-3 PUFAs influence multiple relevant molecular pathways associated with important and diverse roles in cellular and organelle membrane structure and function, tissue metabolism, and genetic regulation. Incorporation of *ω*-3 PUFAs into cellular and organelle membranes influences membrane fluidity of lipid rafts that modulate protein function and signaling events. For example, ion channels such as sodium (Na^+^), L-type calcium (Ca^2+^), and Na^+^−Ca^2+^ exchangers can be modulated by *ω*-3 PUFAs incorporation into lipid membranes. Moreover, *ω*-3 PUFAs are natural ligands of many key nuclear receptors in multiple tissues, and also alter function of several transcription factors.^61^ This molecular regulation contributes to observed effects of *ω*-3 PUFAs on lipid metabolism and inflammatory pathways. An adequate intake of LC *ω*-3 fatty acids, specifically eicosapentaenoic (EPA, 20:5n-3) and docosahexaenoic (DHA, 22:6n-3) acids has been associated with lower incidence of CVD.^62^ Moreover, LC *ω*-3 PUFAs EPA and DHA are metabolized to resolvins and protectins, which are very potent anti-inflammatory mediators in cellular and animal model systems (Fig. 1). Although recent clinical trials have failed to demonstrate a beneficial effect of LC *ω*-3 supplementation on CVD, suggesting that fish is the advisable source of LC *ω*-3 PUFAs, many studies including clinical trials and meta-analysis have concluded that consumption of fish, fish oils, or individual LC *ω*-3 fatty acids is an effective dietary strategy to reduce CVD morbidity, mortality, and risk factors.^63–68^ Recently, the PREDIMED trial reported that in participants without previous CVD and high fish consumption, dietary ALA, supplied mainly by walnuts and olive oil, relates inversely to all-cause mortality, whereas protection from cardiac mortality is limited to fish-derived LC *ω*-3 PUFAs.^69^ However, LC *ω*-3 PUFAs have been shown to improve a number of cardiac parameters such as BP, heart rate, and endothelial function.^70–73^ The cardioprotective effects of LC *ω*-3 PUFAs also include platelet aggregation inhibition, reduction of triglyceride levels, arrhythmia prevention, vascular relaxation improvement, anti-inflammatory responses, enhancement of plaque stability, and antiatherosclerotic effects.^74–76^ In particular, the greatest effect of LC *ω*-3 fatty acids appears to be on triglycerides and is mainly due to a reduction in hepatic very-LDL-cholesterol synthesis. The mechanisms include increased fatty acid *β*-oxidation, increased hepatic synthesis of phospholipids instead of triglycerides, altered enzymatic activity for triglyceride assembly in the liver, and reduced fatty acid availability for triglyceride synthesis as a result of decreased *de novo* lipogenesis.^74^ To date, evidence supports that an adequate intake of LC *ω*-3 PUFAs, either from fatty fish or from supplements, if continued for decades may contribute to reduce the risk for CVD.^63^

## The Importance of *ω*-6/*ω*-3 Fatty Acid Ratio in Cardiovascular Inflammation

In the diet of our ancestors, the ratio of *ω*-6 to *ω*-3 fatty acids was 1 to 2/1 with higher levels of EPA, DHA, and AA.^77^ One of the most important consequences of the consumption of EPA and AA is to be included in plasma membrane phospholipids, particularly of platelets, erythrocytes, neutrophils, and monocytes. Today, the Western diet provides an *ω*-6 to *ω*-3 ratio of around 16:1, indicating a depletion of *ω*-3 fatty acids.^78^ A balanced *ω*-6 to *ω*-3 PUFAs ratio may have a protective effect on age-related diseases, including CVD. Several studies have been conducted to determine the importance of the dietary *ω*-6/*ω*-3 fatty acids ratio, rather than the level of individual PUFAs, in CVD prevention.^79^ Moreover, there is a metabolic competition between *ω*-6 and *ω*-3 since C18 precursor enzymes compete on the same enzymes for elongation and desaturation. It is noteworthy that *ω*-6 and *ω*-3 exert opposite physiological function, therefore an imbalance in *ω*-6/*ω*-3 ratio may result in altered gene expression and disequilibrium in cell membrane composition and fluidity.^80^ The *ω*-6 and *ω*-3 fatty acids regulate and interact with multiple transcription factors and nuclear receptors such as peroxisome proliferator-activated receptors (PPARs), NF-*κβ*, and sterol regulatory element binding protein (SREBP), all of which influence inflammatory responses and lipid metabolism.^81^ The anti-inflammatory actions of eicosanoids from LC *ω*-3 PUFAs are crucial to induce cardioprotective effects. Eicosanoids produced from C20 fatty acids are less inflammatory than their AA-derived eicosanoid counterparts and serve as vasodilators and inhibitors of platelet aggregation.^82^ The LC *ω*-3 PUFAs can reduce the production of AA-derived eicosanoids by competing with AA. Therefore, high intake of dietary *ω*-6 PUFAs increases the synthesis of proinflammatory eicosanoids derived from AA, and inhibits the synthesis of anti-inflammatory eicosanoids from EPA.^79^ Long-term production of proinflammatory eicosanoids from AA and high *ω*-6 PUFAs dietary intakes may increase the risk of heart disease and contribute to the development of CVD. In contrast, a higher consumption of LC *ω*-3 PUFAs in the diet results to have a favorable effect on *ω*-6/*ω*-3 ratio and promote anti-inflammatory properties through the reduction of proinflammatory eicosanoid synthesis from monocytes, neutrophils, eosinophils, platelets, and endothelial cells.^83–86^

## CPs and Reduction of *ω*-6/*ω*-3 Fatty Acid Ratio: Potential Implications on Cardiovascular Health

**FIG. 2.**
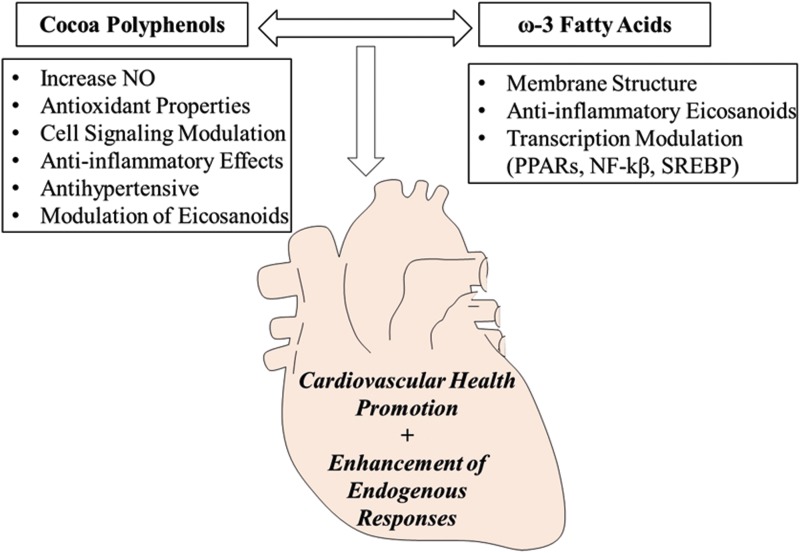
Proposed nutritional strategy to reduce cardiovascular risk factors. This model links the combined use of CPs and *ω*-3 fatty acids to cardiovascular health promotion. The figure represents a summary of the mechanisms that could potentially contribute to prevent or reduce the onset of age-related cardiovascular events and enhance cardioprotective responses. CPs, cocoa polyphenols.

The crucial role of *ω*-3 PUFAs in the diet is evident, and the need to return to a more physiologic *ω*-6 to *ω*-3 ratio of 2:1 rather than the ratio of 16:1 provided by current Western diets. Experimental studies have provided evidence that dietary supplementation of LC *ω*-3 PUFAs modifies inflammatory and immune reactions, suggesting LC *ω*-3 PUFAs as potential therapeutic agents for inflammatory diseases.^87^ The metabolism of eicosanoids represents a novel target for the prevention or treatment of inflammation in CVD.^88,89^ The eicosanoid family includes derivatives of C20 PUFAs, mainly AA. This *ω*-6 fatty acid is released from the phospholipids through the activity of phospholipase A2 (PLA2) enzymes, which are stimulated by inflammatory signals. Then, AA acts as a substrate for COX and LOX. COX enzymes lead to PGE2 and TxA2, LOX enzymes to LT4. Eicosanoids are recognized as crucial mediators of both physiological and pathophysiological responses of the microcirculation and also as key regulators of inflammation. These molecules play a crucial role on vascular permeability and tone and in the recruitment of various inflammatory cells from the circulation into tissues. It is important to highlight again that the conversion of ALA to EPA is in competition with the conversion of LA to AA, since the same enzymes are involved (Fig. 1). The Δ-6 desaturase reaction is rate limiting in this pathway. Although the optimal substrate for Δ-6 desaturase is ALA, LA is prevalent in human diets than ALA; therefore, metabolism of *ω*-6 fatty acids is largely preferred. The *ω*-6-PUFA AA is the primary source of fatty acids and elevated tissue levels of AA have been associated with inflammatory disease states, including obesity and CVD.^90,91^ However, LC *ω*-3 fatty acids EPA and DHA inhibit AA metabolism, and EPA induces eicosanoid mediators such as PGE3, TxA3, and LT5 that may block the activity of those derived from AA.^92^ The anti-inflammatory properties of LC *ω*-3 PUFAs, especially EPA, are due to competition with AA as substrate for COX and 5-lipoxygenase (5-LOX). To date, there are substantial data that EPA and DHA are able to inhibit several aspects of inflammation such as production of eicosanoids like PGE2 and LT4 from the *ω*-6 fatty acid AA. To improve the ratio of *ω*-6 to *ω*-3 PUFAs and reduce the production of proinflammatory eicosanoids, it is necessary to decrease the intake of *ω*-6 PUFAs from vegetable oils and increase the intake of LC *ω*-3 PUFAs by using oil fish and fish oil supplements.^93^ Interestingly, consumption of polyphenols from red wine has been associated with increased levels of EPA and DHA in patients with heart disease, a phenomenon referred to as a “fish-like” effect.^94^ In this context, although little research has been done to support the role of CPs in modulating eicosanoid metabolism, *in vitro* studies have shown that CPs are potent inhibitors of eicosanoid-generating enzymes, and can suppress the production of inflammatory eicosanoid metabolites.^95,96^ Although the effect of CPs on the AA metabolism remains to be completely defined, Gu *et al.* found that cocoa-supplemented mice had 33% lower levels of AA compared with high-fat diet-fed mice. Furthermore, cocoa treatment also reduced availability of substrates for the synthesis of eicosanoids, particularly the protein levels of PLA2 and (COX-2).^97^ An imbalance in PGE2/TxA2 production has been linked to CVD and a short-term investigation reported that chocolate procyanidins and cocoa flavanols decrease the ratio of PGE2/LT4 and PGE2/TxA2 in humans, supporting the hypothesis that cocoa-derived flavonoids can favorably alter eicosanoid synthesis in humans.^98,99^ From these data, it is possible to support the concept that CPs can modulate eicosanoid profiles and possibly reduce the detrimental effects of elevated intakes of *ω*-6 fatty acids at the level of LA and AA. Moreover, a combination treatment with CPs and LC *ω*-3 fatty acids may be a novel and effective rationale for future studies investigating the protective role of CPs and PUFAs. This rationale, depicted in Figure 2, provides a biochemical and clinical framework for pursuing clinical trials evaluating this combination as a nutritional approach to prevent CVD. Furthermore, it also reasonable to speculate that the combined effects of CPs and *ω*-3 PUFAs may decrease the attenuation of endogenous protective mechanisms associated with the aged heart. A hypothesis regarding the effect of polyphenols on LC *ω*-3 PUFAs metabolism is that they may accelerate the synthesis of EPA and DHA from their precursor ALA. Whether polyphenols influence Δ-6 and Δ-5 desaturases requires further studies, however, some classes of polyphenols have been linked at least partly to a PPAR*α*-dependent mechanism that interferes with the PUFA elongation-desaturation pathway.^100–102^ Although the exact mechanisms involved in the interactions between polyphenols and PUFAs are not yet understood, recent data show that dietary flavonoids interact with the metabolism of LC *ω*-3 PUFAs and increase blood EPA and DHA levels.^103^

## Conclusions

Nutritional strategies for enhancing cardioprotection and reducing CVD risk will continue to be an important area of research. For instance, the ongoing Cocoa Supplement and Multivitamin Outcomes Study (COSMOS), which aims to determine the efficacy of a flavanol-rich cocoa using a 5-year randomized trial among 18,000 healthy men and women, may provide definitive evidence on the health benefits of cocoa on cardiovascular outcomes.^104^ Based on the existing literature discussed here, it is evident that CPs and LC *ω*-3 PUFAs play crucial roles in the prevention of CVD. Many studies have shown the individual cardioprotective nature of these compounds, however, more efforts should be directed to understand whether a synergistic combination of CPs and LC *ω*-3 PUFAs is more beneficial for CVD prevention. Despite the paucity of studies assessing the synergistic benefits of CPs and LC *ω*-3 PUFAs on CVD, there is a growing body of evidence that can serve as a translational platform to design future human studies and address questions behind the fascinating outcomes of a combined approach using CPs and LC *ω*-3 fatty acids.^105–110^ There are interesting preliminary results in rodent studies that encourage further work in this field and which hold promise for utilizing a combined use of CPs and LC *ω*-3 fatty acids as a preventive tool in CVD. Although clinical/translational evidence already supports nutraceutical compounds for healthy aging, alternative strategies with combined actions of food bioactive compounds have the potential to enhance cardioprotection and cardiovascular health in older adults for whom a single compound may be not enough. Clearly, more work is needed to provide novel insights into the mechanisms by which CPs and LC *ω*-3 fatty acids synergize to prevent and/or reduce cardiovascular risk factors. However, the co-administration of these agents may be a possible nutraceutical strategy for a number of disorders that affect the heart or blood vessels.
